# Diet supplementation with green tea extract epigallocatechin gallate prevents progression to glucose intolerance in *db/db *mice

**DOI:** 10.1186/1743-7075-9-11

**Published:** 2012-02-14

**Authors:** Henrik Ortsäter, Nina Grankvist, Swen Wolfram, Nicolas Kuehn, Åke Sjöholm

**Affiliations:** 1 Karolinska Institutet, Department of Clinical Science and Education, Södersjukhuset, SE-118 83 Stockholm, Sweden; 2 DSM Nutritional Products Ltd, Department of Human Nutrition and Health, P.O. Box 2676, Bldg.241/958, CH-4002 Basel, Switzerland; 3 Diavet Labor, Schlyffistrasse 10, CH-8806, Bäch/SZ Zurich, Switzerland

**Keywords:** Green tea, Epigallocatechin gallate, Diabetes islet, Beta cell, Insulin secretion

## Abstract

**Background:**

Green tea was suggested as a therapeutic agent for the treatment of diabetes more than 70 years ago, but the mechanisms behind its antidiabetic effect remains elusive. In this work, we address this issue by feeding a green tea extract (TEAVIGO™) with a high content of epigallocatechin gallate (EGCG) or the thiazolidinedione PPAR-γ agonist rosiglitazone, as positive control, to *db/db *mice, an animal model for diabetes.

**Methods:**

Young (7 week-old) *db/db *mice were randomized and assigned to receive diets supplemented with or without EGCG or rosiglitazone for 10 weeks. Fasting blood glucose, body weight and food intake was measured along the treatment. Glucose and insulin levels were determined during an oral glucose tolerance test after 10 weeks of treatment. Pancreata were sampled at the end of the study for blinded histomorphometric analysis. Islets were isolated and their mRNA expression analyzed by quantitative RT-PCR.

**Results:**

The results show that, in *db/db *mice, EGCG improves glucose tolerance and increases glucose-stimulated insulin secretion. EGCG supplementation reduces the number of pathologically changed islets of Langerhans, increases the number and the size of islets, and heightens pancreatic endocrine area. These effects occurred in parallel with a reduction in islet endoplasmic reticulum stress markers, possibly linked to the antioxidative capacity of EGCG.

**Conclusions:**

This study shows that the green tea extract EGCG markedly preserves islet structure and enhances glucose tolerance in genetically diabetic mice. Dietary supplementation with EGCG could potentially contribute to nutritional strategies for the prevention and treatment of type 2 diabetes.

## Introduction

The WHO and CDC (U.S. Center for Disease Control) predict that by today some 26 million people in the U.S. only are afflicted by diabetes (http://www.cdc.gov/diabetes/). Previously viewed as a disease of the elderly, type 2 diabetes is now seen in ever-younger age groups. In the U.S. about one third of all newly diagnosed diabetes in children and adolescents (age 10-19 years) now is type 2, an alarming scenario considering the magnitude of premature cardiovascular and cerebrovascular morbidity in these individuals. Recent estimates by the CDC indicate that the life-time risk of getting diabetes is not less than 40% for people born in 2000 in the U.S., with certain ethnic groups being significantly overrepresented (http://www.cdc.gov/diabetes). In the U.S. alone, the annual cost for diabetes care is $174 billion, of which 97% is targeted to type 2 diabetes (http://www.cdc.gov/diabetes).

Second only to water, tea is the most consumed beverage in the world and its beneficial properties have been widely explored. Green tea has for centuries been used in folk remedy to treat a number of ailments such as diabetes. However, the precise mechanisms by which green tea exerts its salutary effects remain unknown. The tea leaf is rich in tea polyphenols, accounting for 25-35% of the dry weight. The flavonoid EGCG (epigallocatechin gallate) is quantitatively the most important one and is considered the main active ingredient of green tea [1]. Over the past few decades, there has been growing interest in EGCG, because it has been suggested to exert an array of beneficial cardiovascular influences [2-6]. The exact mechanism of action of EGCG with regard to these various effects is largely unknown and in many cases is assumed to be due to its antioxidant activity.

It was previously shown that EGCG ameliorates cytokine-induced β cell damage in vitro [7] and prevents the decrease of islet mass induced by treatment with multiple low doses of streptozotocin (STZ) *in vivo *[8]. However, in the latter study STZ was co-injected with EGCG, which possesses strong antioxidative activity [9]. It is unclear whether the protective effects observed in this study were due to direct inactivation of the co-injected STZ. In contrast to these studies indicating an antioxidative capacity of EGCG, investigations in the insulinoma cell line HIT-T15 showed that EGCG treatment was associated with increased production of reactive oxygen species and reduced cell viability [10].

Thus, the antidiabetic effects of EGCG are not entirely clarified. The *in vivo *relevance of potentially antidiabetic green tea catechins remains to be demonstrated, particularly with regard to β cell function. Therefore, we conducted an *in vivo *study in the *db/db *mouse to explore the antidiabetic effects of dietary supplementation with a pharmacological dose of the most abundant green tea catechin, EGCG. In addition, we have also performed *ex vivo *analysis of pancreatic islets and MIN6 cells treated with palmitate in combination with EGCG. The reported results show that, by adding EGCG to diet, the progression to glucose intolerance in *db/db *mice can be prevented.

## Material and methods

### Animal care

The present study was approved by the local Animal Ethics Committee. The investigation conformed to the National Institutes of Health (NIH) *Guide for the Care and Use of Laboratory Animals *[DHHS Publication No. (NIH) 85-23, Revised 1985, Office of Science and Health Reports, Bethesda, MD 20892]. All animals were maintained on a 12 h light (300 Lux) and 12 h dark cycle at a humidity of 55-60% and a temperature of 23 ± 1°C. All *db/db *animals received modified AIN-93 diets (Provimi Kliba AG, Kaiseraugst, Switzerland) [11] and water ad libitum.

### Experimental design *in vivo*

The effect of dietary EGCG (TEAVIGO™, DSM Nutritional Products Ltd, Basel, Switzerland) supplementation on type-2-like diabetes was investigated by utilizing the *db/db *diabetic mouse model (BKS.Cg m^+/+ ^Lepr^db^). TEAVIGO™ is a highly purified extract from green tea leaves (*Camellia sinensis*) containing > 94% EGCG, < 5% other catechins (< 3% epicatechin gallate). Male (7 weeks old) *db/db *mice (Jackson Laboratories, Bar Harbor, ME) were randomized to receive placebo diet, a modified AIN-93 diet containing EGCG at a concentration of 10 g/kg of diet (EGCG 1% [w/w]) or a diet containing the thiazolidinedione rosiglitazone (Avandia™, GlaxoSmithKline, Brentford, UK) at a concentration of 21 mg/kg of diet (Rosi 0.0021% [w/w]) for 10 weeks. Fasting (2 hour) blood glucose levels were measured at 0, 5 and 10 weeks, food intake and body weight were monitored at 0, 3, 6 and 9 weeks. After 10 weeks of dietary treatment, an oral glucose tolerance test (OGTT) was performed. Before application of an oral glucose load (1 g/kg, Sigma, St. Louis, MO), blood glucose levels were determined in food-deprived animals (Glucotrend, Roche Diagnostics, Basel, Switzerland). Plasma insulin was determined by use of an ELISA kit (Mercodia AB, Uppsala, Sweden). Insulin resistance was assessed by either homeostasis model assessment-estimated insulin resistance (HOMA-IR) = fasting glucose (mM) * fasting insulin (μU/ml)/22.5, or by quantitative insulin sensitivity check (QUICKI) = 1/[log ((fasting glucose (mg/dl) + (fasting insulin (μU/ml))]. After the OGTT, animals were sacrificed and pancreas was taken for further experiments.

### Pancreas histology and insulin content

The pancreas of each animal was carefully dissected, a small part of the splenic part of the gland was weighed and placed in acid ethanol (0.18 M HCl in 95% ethanol) to determine insulin content and the rest was immersed in formalin solution and stored at 4°C until further processing for histological examination. Preparations were fixed in 4% buffered neutral formalin, embedded in paraffin and cut at 4 μm. The pancreas was cut on three different levels (each 100 μm apart) for both the splenic and the duodenal part to get a representative overview. So in total, there were 6 measurements that were averaged. A part of the sections was stained with hematoxylin and eosin (HE). The other sections were immunolabeled with antiinsulin antibodies. For this purpose, sections were deparaffinized and re-hydrated, and then incubated for 25 minutes in 70% methanol and hydrogen peroxide (H_2_O_2_). After washing with Tris-buffer-saline (TBS, pH 7.3), the sections were incubated overnight at 4°C with an antiinsulin antibody (Serotec, Inotech AG, Dottikon, Switzerland) in TBS + 10% bovine serum, dilution 1:1000 and then for 90 minutes with an antiguinea pig antibody (Vectastain, Vector, Reactolab SA, Servion, Switzerland) in TBS + 10% bovine serum, dilution 1:200. After washing, sections were incubated with avidin-peroxidase complex (Vectastain, Vector, Reactolab SA, Servion, Switzerland) for 150 minutes and then washed again. The sections were stained with 3,3 diaminobenzidin (DAB) for 5 minutes and counter-stained with hemalum (Mayer) for 45 seconds.

On each section, the total number of islets and the relative number of pathological islets (in %) were determined. Pathological islets were defined by islet atrophy due to loss of islet cells. This is histologically recognizable as an abnormally small size and shrinkage of the islet, characterized by loss of definition of islet boundaries and displacement of exocrine tissue (single cells, acini, ducts) into the islet tissue. Evaluation was performed with a Nikon Eclipse E400 microscope (Nikon AG, Egg, Switzerland).

The relative number of islets (per cm^2 ^of pancreas), average islet size (in μm^2^), relative endocrine area (in % of the pancreatic surface), and relative β cell area (in % of the whole endocrine surface) were determined with software (program Stereo Investigator, Williston, VT). The different areas were assessed with the Cavalieri method. Briefly, this method allows estimation of a surface area with the help of a grid placed over that surface. The surface to be evaluated is divided in multiple squares of equal size by this way. The surface area to be estimated is then equal to the number of intersection points of the lines of the grid which hit that surface, multiplied by the area of one square. The smaller the squares are chosen, and the higher the number of intersection points, the more accurate the estimation will be and the closer to the real size of the surface. Histological pictures were taken with a Nikon digital camera DXM 1200 (Nikon AG, Egg, Switzerland). All evaluations were performed by an independent observer blinded to the treatment of the animals.

Pancreas insulin content was measured after neutralization with an ELISA kit (Mercodia AB, Uppsala, Sweden).

### Culture of isolated islets and MIN6 cells

For *ex vivo *studies, 6 months old male C57Bl/6J mice obtained from our local breed were used. Pancreatic islets were isolated by collagenase digestion. Individual islets were handpicked and placed in RPMI 1640 culture medium (SVA, Uppsala, Sweden) containing 11 mM glucose and supplemented with 10% FBS, 2 mM L-glutamine and 60 μg/ml penicillin G and 50 μg/ml streptomycin sulfate for an overnight recovery at 37°C and 5% CO_2_. On the next day, islets were transferred to same type of media but with 1% FBS and in the absence or presence of 0.5 mM palmitate complexed with 0.5% fatty acid free BSA (Boehringer Mannheim GmbH, Mannheim, Germany) and with or without 5, 10 or 20 μM EGCG (prepared from 20 mM stock dissolved in DMSO). Islets were exposed for 24 hours.

MIN6 cells (22), derived from mouse pancreatic β cells, were maintained in Dulbecco's Modified Eagle Medium containing 25 mM glucose and sodium pyruvate supplemented with 15% FBS, 6 mg/ml penicillin G, 5 mg/ml streptomycin sulfate (Invitrogen Inc., Carlsbad, CA), 2 mM L-glutamine (SVA, Uppsala, Sweden) and 50 μM β-mercaptoethanol at 37°C and 5% CO_2_. During palmitate exposure, media was supplemented with 0.5 mM palmitate and 0.5% fatty acid-free BSA.

### Islet and MIN6 mRNA analyses

Islets were isolated, by collagenase digestion of pancreata (see above), from *db/db *mice after 10 weeks of dietary placebo, rosiglitazone or EGCG supplementation. Islets from 9 mice in each group were pooled into 4 independent samples. After isolation, islets were washed twice in PBS and then total mRNA was extracted using Aurum™ Total RNA Mini kit (Bio-Rad, Hercueles, CA) according to the manufacturer's instructions and reversely transcribed with iScript™ cDNA Synthesis kit (Bio-Rad). Quantitative real-time PCR was performed in 20 μl volume containing ~25 ng cDNA, 0.5 μmol/l forward and reverse primers and 10 μl iQ™ SYBR^® ^Green Supermix (Bio-Rad). RNA isolation, cDNA synthesis and qPCR of MIN6 exposed to palmitate in the absence or presence of EGCG were performed as with islets. Primers used for the amplification are shown in Table 1. PCR products were quantified fluorometrically using SYBR Green and normalized to the housekeeping gene β-actin and relative to the placebo group [12].

**Table 1:** Analysis of islet and MIN6 cell mRNA expression

	*db/db *islets			MIN6 cells					
**Gene name**	**Control**	**Rosi**	**EGCG**	**Control**	**EGCG**	**Palmitate**	**EGCG+Palmitate**	**Forward**	**Reverse**

Cpt-1	100 ± 21	36 ± 12*	25 ± 9*	100 ± 10	79 ± 3	1182 ± 90*	615 ± 46#	5'-GATCTACAATTCCCCTCTGC-3'	5'-CCTCTGTGGTACACGACAAT-3'

Glut-2	100 ± 10	78 ± 19	70 ± 24	100 ± 2	102 ± 9	33 ± 1*	32 ± 3	5'-TGGCTTTCACTGTCTTCACT-3'	5'-GTGCCATTGACGTCATAGTT-3'

Glucokinase	100 ± 9	123 ± 5	83 ± 10	100 ± 5	88 ± 13	89 ± 5	60 ± 8	5'-GGCACGAAGACATAGACAAG-3'	5'-CACCACATCCATCTCAAAGT-3'

Ddit3	100 ± 19	111 ± 16	61 ± 5*	100 ± 19	74 ± 14	646 ± 25*	485 ± 73#	5'-TCTGTCTCTCCGGAAGTGTA-3'	5'-CTGGTCTACCCTCAGTCCTC-3'

Ppp1r15a	100 ± 7	98 ± 4	71 ± 5*	100 ± 6	91 ± 7	1004 ± 57*	628 ± 50#	5'-TTCTATCTCCTGTCCCCACT-3'	5'-TACCAGAGACAGGGGTAGGT-3'

Cdkn1a	100 ± 23	88 ± 19	43 ± 11*	100 ± 18	150 ± 43	439 ± 51*	579 ± 26	5'-ATTGCTCAGACCTGTGAAGA-3'	5'-AGCAGCAGATCACCAGATTA-3'

Pdx1	100 ± 16	90 ± 8	86 ± 10	-	-	-	-	5'-CCCTGAGCTTCTGAAAACTT-3'	5'-AGCCCAGGTTGTCTAAATTG-3'

Insulin 1	100 ± 15	126 ± 23	58 ± 10*	100 ± 3	103 ± 5	64 ± 4*	51 ± 2#	5'-AGGTAGGCAACCGTGTAAAT-3'	5'-ACCTTCCTCTCAGGAGTCAG-3'

Glucagon	100 ± 11	216 ± 30*	46 ± 7*	-	-	-	-	5'-GATTTTGTGCAGTGGTTGAT-3'	5'-ACTTCTTCTGGGAAGTCTCG-3'

Somatostatin	100 ± 13	116 ± 21	39 ± 4*	-	-	-	-	5'-TCTGGAAGACATTCACATCC-3'	5'-CAATTTCTAATGCAGGGTCA-3'

Analysis of mRNA expression in islets of Langerhans isolated from control *db/db *mice and *db/db *mice receiving dietary supplementation with 1% (w/w) EGCG or 0.0021% (w/w) rosiglitazone (Rosi) for 10 weeks. The mRNA levels of the indicated genes were normalized to the levels of β-actin mRNA and expressed as mean percent ± SEM of the control group, n = 4. * denotes *P *< 0.05 for a chance difference vs controls and # denotes *P <*0.05 for a chance difference vs palmitate using one-way ANOVA. MIN6 cells were exposed in vitro for 48 hrs to EGCG (20 μM), palmitate (0.5 mM) or a combination thereof

### Assessment of cell viability and apoptosis

Cell viability was assayed with the Cytotoxicity Detection Kit (Plus Roche Diagnostics GmbH, Mannheim, Germany). The assay measures the amount of lactate dehydrogenase released from cells after lysis, which correlates inversely to the amount of live cells after treatment. Apoptosis was assayed with the cell death detection kit ELISA^PLUS ^(Roche Diagnostics GmbH, Mannheim, Germany). The ELISA measures cytoplasmic oligonucleosomes that increase after apoptosis-associated DNA degradation.

### Western blot analysis

Samples of total protein extracted from untreated and treated islets or MIN6 cells were subjected to SDS-PAGE (15-20 μg protein per sample). After electrophoresis, proteins were transferred onto PVDF membranes. Immunoblot analyses were performed with antibodies against phosphorylated JNK 1/2, total JNK1/2 and the cleaved form of caspase 3 (all obtained from Cell Signaling Inc.). Immunoreactive bands were developed using ECL, imaged with a GelDoc system and quantified with Quantity One software (Bio-Rad). After imaging, to verify equal protein loading, the PVDF membranes were stained with Coomassie.

### Statistical analysis

All data are expressed as means ± SEM for animals in each group. Statistical significance of the mean differences between groups was tested by one-way analysis of variance (ANOVA). *P *values less than 0.05 were considered significant.

## Results

### Blood glucose, food intake and body weight during *10 weeks *of treatment with EGCG or rosiglitazone

Data on fasting blood glucose, accumulated food intake and body weight during the diet treatment of *db/db *mice is given in Table 2. Fasting blood glucose levels in 7 weeks old *db/db *mice were approximately 5 mM prior to treatment. During the 10 week treatment period fasting blood glucose levels rose 3-fold in control mice ending at 14.7 mM. Significantly lower fasting blood glucose levels were seen in mice receiving either EGCG or rosiglitazone after 10 weeks of treatment. In control mice body weight increased from 21.9 to 46.0 gram during the study period. Mice having a diet supplemented with rosiglitazone gained more weight, ending at 60.4 gram. On the contrary, mice treated with EGCG had a slightly lower body weight (42.2 gram) compared to control mice. There was no difference in food intake when comparing control mice and mice treated with EGCG. Mice receiving rosiglitazone consumed more food compared to control mice.

**Table 2:** Fasting blood glucose, accumulated food intake and body weight during the diet treatment of *db/db *mice

	Fasting blood glucose (mM)			Body weight (gram)				Food intake (gram/day and mouse)		
	**Weeks on diet**			**Weeks on diet**				**Weeks on diet**		

	**0**	**5**	**10**	**0**	**3**	**6**	**9**	**3**	**6**	**9**

Control	5.1 ± 0.6	7.7 ± 0.9	14.7 ± 1.8	21.9 ± 1.4	30.6 ± 0.6	38.7 ± 0.6	46.0 ± 0.7	3.7 ± 0.1	3.5 ± 0.1	3.3 ± 0.1

Rosi	4.9 ± 0.5	4.0 ± 0.3*	6.1 ± 1.0*	24.5 ± 1.0	39.6 ± 0.9*	51.3 ± 0.7*	60.4 ± 0.7*	3.7 ± 0.1	4.2 ± 0.2*	4.0 ± 0.2*

EGCG	5.1 ± 0.4	6.2 ± 0.9	9.3 ± 1.1*	22.7 ± 0.5	30.9 ± 0.5	37.5 ± 0.5	42.2 ± 0.5*	3.6 ± 0.2	3.5 ± 0.1	3.2 ± 0.05

Analysis of fasting blood glucose, body weight and food intake in control *db/db *mice and *db/db *mice receiving dietary supplementation with 1% (w/w) EGCG or 0.0021% (w/w) rosiglitazone (Rosi) for up to 10 weeks. Data is expressed as mean ± SEM for 9 mice in each group. * denotes *P *< 0.05 for a chance difference vs controls using one-way ANOVA in conjunction with Dunnett's multiple comparison test

### Glycemic control and plasma insulin levels after *10 weeks *of treatment with EGCG or rosiglitazone

**Figure 1:**
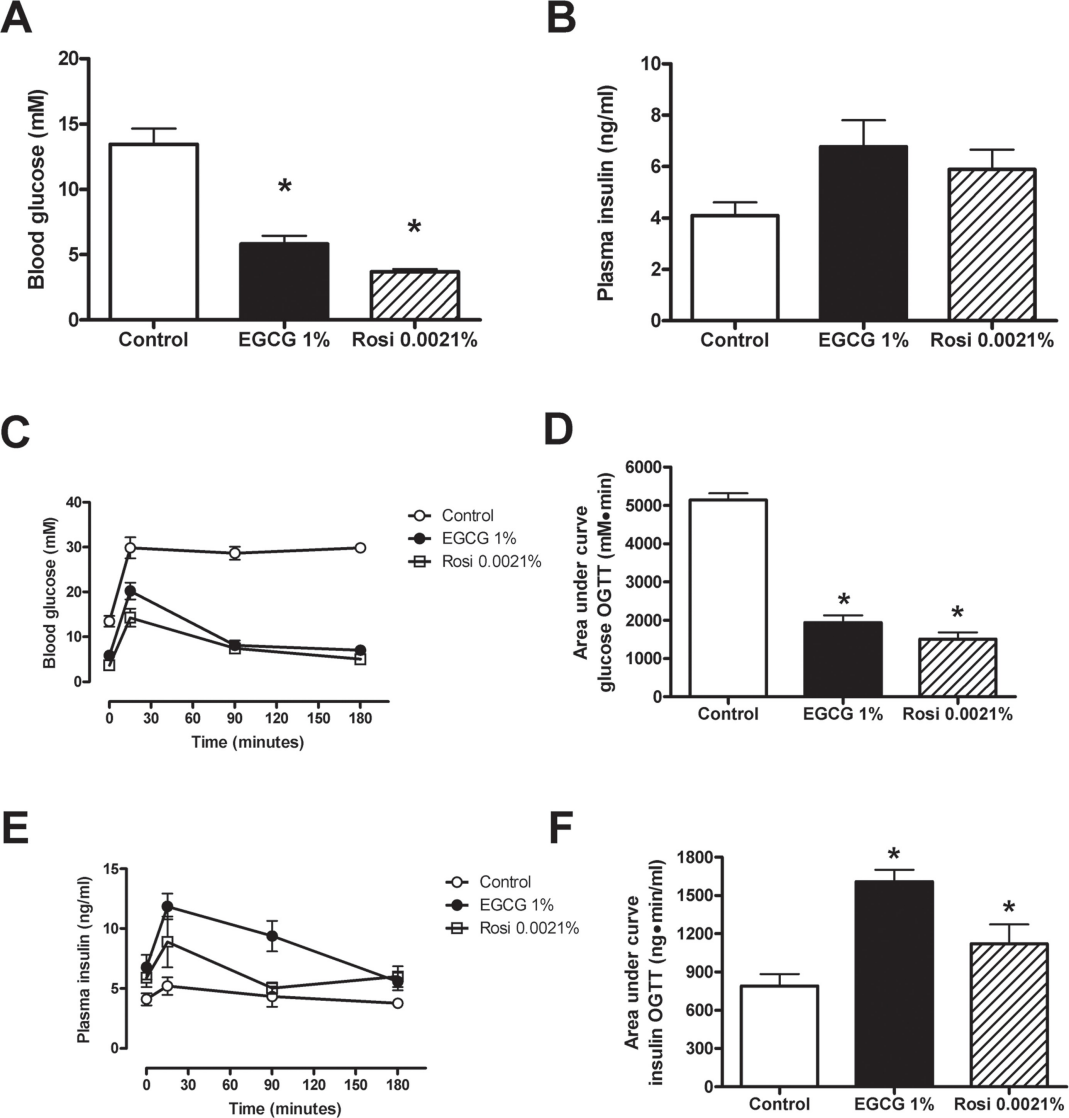
**Improved glycemic control in *db/db *mice after *10 weeks *of treatment with EGCG or rosiglitazone**. Mice received dietary supplementation with 1% (w/w) EGCG or 0.0021% (w/w) rosiglitazone (Rosi) for 10 weeks. Shown are fasting blood glucose levels (**A**), fasting plasma insulin levels (**B**), blood glucose concentrations (**C, D**) and plasma insulin concentrations (**E, F**) during an oral glucose tolerance test (OGTT). Raw data from OGTT are presented in C and E while calculated area under the curve is presented in D and F for blood glucose and plasma insulin respectively. Values represent mean ± SEM for 9 mice in each group. * denotes *P *< 0.05 for a chance difference vs controls using one-way ANOVA in conjunction with Dunnett's multiple comparison test.

**Figure 2:**
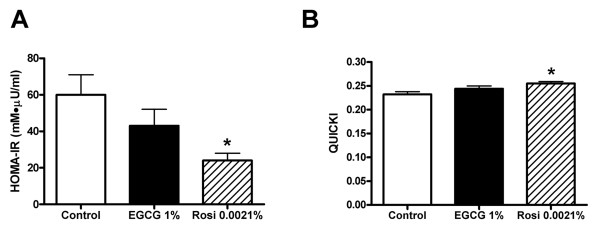
**Changes in insulin resistance after *10 weeks *of treatment with EGCG or rosiglitazone**. Peripheral insulin sensitivity was assessed in control *db/db *mice and *db/db *mice receiving a diet supplemented with 1% (w/w) EGCG or 0.0021% (w/w) rosiglitazone (Rosi) for 10 weeks. Insulin resistance was evaluated by two different means: Shown in A is homeostasis model assessment-estimated insulin resistance (HOMA-IR) that was calculated as fasting glucose (mM) * fasting insulin (μU/ml)/22.5. Quantitative Insulin Sensitivity check (QUICKI) is shown in B and was calculated as 1/[log ((fasting glucose (mg/dl) + (fasting insulin (μU/ml))]. Bars represent mean ± SEM for 9 mice in each group. * denotes *P *< 0.05 for a chance difference vs controls using one-way ANOVA in conjunction with Dunnett's multiple comparison test.

Fasting glycemia, plasma insulin values and glucose and insulin profiles during OGTT are shown in Figure 1 for *db/db *mice treated with EGCG or rosiglitazone for 10 weeks. Animals receiving EGCG or rosiglitazone had lower fasting blood glucose levels (Figure 1A) and but similar fasting plasma insulin levels (Figure 1B) after 10 weeks of treatment with EGCG or rosiglitazone compared with non-treated animals. Glycemic excursions (Figure 1C and 1D) during the OGTT were significantly smaller in animals receiving EGCG or rosiglitazone than in control mice, and essentially normalized at the end of the OGTT. Conversely, insulin responses (Figure 1E and 1F) to the OGTT were enhanced in EGCG- or rosiglitazone-treated animals. When insulin sensitivity was assessed (Figure 2A and 2B, respectively), it was evident that rosiglitazone enhanced insulin sensitivity significantly following 10 weeks of oral treatment. In contrast, EGCG failed to significantly improve insulin sensitivity after 10 weeks of treatment (Figure 2).

### Islet histomorphometry

**Figure 3:**
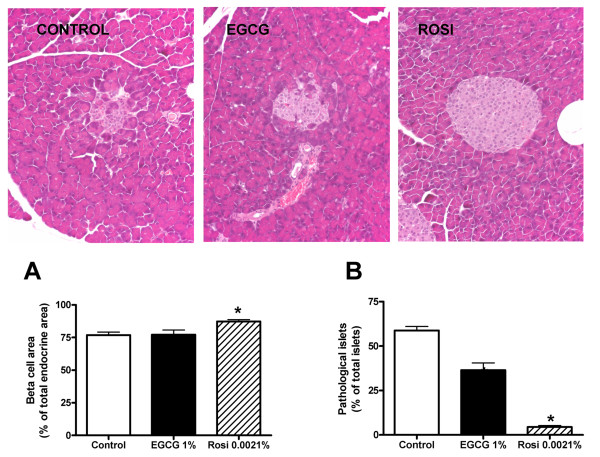
**Effects of *10 weeks *of treatment with EGCG or rosiglitazone on pancreatic islet morphology and β cell area**. Light microscopic appearance and histomorphometric analysis of pancreatic islet morphology and β-cell area in control *db/db *mice and *db/db *mice receiving dietary supplementation with 1% (w/w) EGCG or 0.0021% (w/w) rosiglitazone (Rosi) for 10 weeks. H&E staining was used and original magnification was 200 x. Bars represent mean ± SEM for 9 mice in each group. * denotes *P *< 0.001 (number of pathological islets) and *P *< 0.01 (β cell area) for chance differences vs controls using one-way ANOVA in conjunction with Dunnett's multiple comparison test.

**Figure 4:**
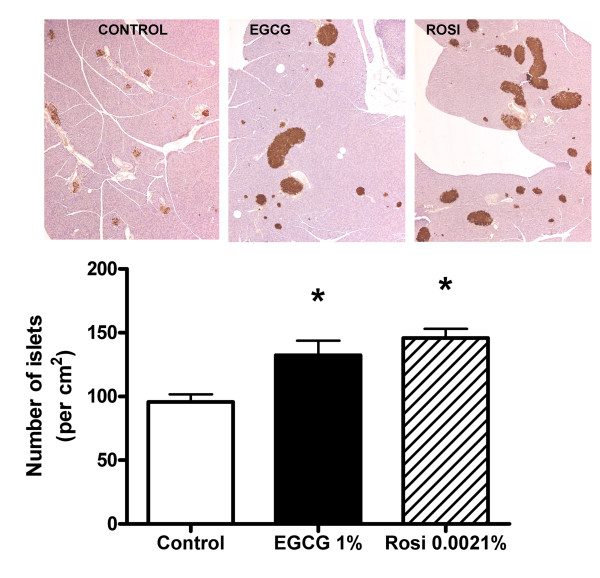
**Increased number of pancreatic islets after *10 weeks *of treatment with EGCG or rosiglitazone**. Light microscopic appearance of pancreatic islets immunostained for insulin in control *db/db *mice and *db/db *mice receiving dietary supplementation with 1% (w/w) EGCG or 0.0021% (w/w) rosiglitazone (Rosi) for 10 weeks. Islets are immunostained for insulin (brown) and original magnification was 100 x. Bars represent mean ± SEM for 9 mice in each group. * denotes *P *< 0.05 for a chance difference vs controls using one-way ANOVA in conjunction with Dunnett's multiple comparison test.

**Figure 5:**
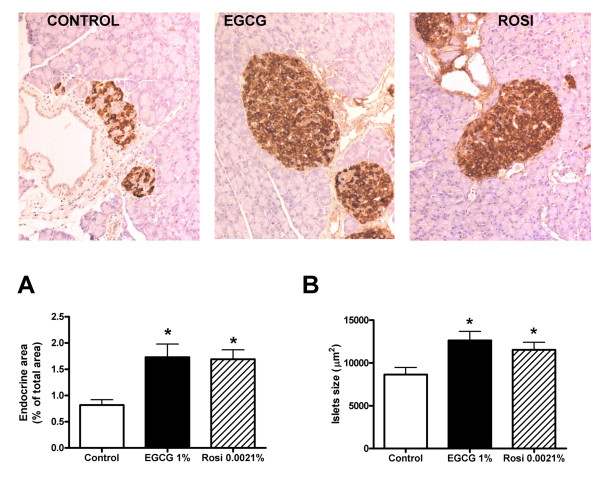
**Increased size and endocrine area of pancreatic islets after 10 weeks of treatment with EGCG or rosiglitazone**. Light microscopic appearance of pancreatic islets immunostained for insulin in control *db/db *mice and *db/db *mice receiving dietary supplementation with 1% (w/w) EGCG or 0.0021% (w/w) rosiglitazone (Rosi) for 10 weeks. Islets are immunostained for insulin (brown) and original magnification was 200 x. Bars represent mean ± SEM for 9 mice in each group. For islet size, * denotes *P *< 0.001, and for endocrine area *P *< 0.01, for chance differences vs controls using one-way ANOVA in conjunction with Dunnett's multiple comparison test.

During progression to diabetes islet structure degenerate [13]. To evaluate if EGCG and/or rosiglitazone were able to prevent islet degeneration in the *db/db *mouse model we used light microscopy to examine islet structure after insulin immunostaining. As shown in Figure 3, 10 weeks of EGCG treatment caused a significant although not to same extent as rosiglitazone, decrease in the number of islets showing pathological changes. Treatment with EGCG for 10 weeks did not significantly influence the size of the β cell area relative to the total endocrine area in islets, whereas rosiglitazone modestly increased β cell area. After 10 weeks of EGCG treatment there was a significant increase in the number of islets per area of pancreas, of comparable magnitude to that evoked by rosiglitazone (Figure 4). Lastly, as shown in Figure 5, 10 weeks of EGCG treatment caused a significant increase in the size of the pancreatic islets, again of comparable magnitude to that evoked by rosiglitazone. Together with the hyperplastic effects of the substances noted in Figure 4, this gave rise to > 2-fold increase in pancreatic endocrine area. The presence of isolated, invasive acinar cells among the endocrine cells in the islets from untreated mice (Figure 5, left panel) suggests that these are degenerated islets. Note the sparsely granulated endocrine and heavily granulated acinar cells located within the degenerate islets. In contrast, note the pronounced granulation of the β cells and the apparently normal morphology of islets from mice treated with EGCG (middle) and rosiglitazone (right).

Pancreatic insulin content in control *db/db *mice was 0.9 ± 0.1 U insulin per gram pancreas wet weight. Diet treatment with EGCG or rosiglitazone increased pancreatic insulin content to 1.6 ± 0.1 U/gram and 1.9 ± 0.1 U/gram, respectively. Although these increases were not statistically significant (*P *= 0.09, N = 9), these observations are nonetheless consistent with the histological data.

### Islet mRNA levels after *10 weeks *of treatment with EGCG or rosiglitazone

Quantitative real-time RT-PCR was used to analyze mRNA expression of genes involved in glucose sensing, endoplasmic reticulum stress, cell proliferation and genes for pancreatic hormones in islets derived from *db/db *mice fed a control diet or diets supplemented with either EGCG or rosiglitazone (Table 1). Islet mRNA expression of Glut-2 and glucokinase was not affected by EGCG or rosiglitazone. On the other hand, both supplements caused an approximately 65% reduction in islet L-CPT-1 mRNA levels. We also measured mRNA levels of endoplasmic reticulum (ER) stress-associated genes, whose levels are reported to be up-regulated in the *db/db *mice [14,15]. Dietary supplementation with EGCG, but not rosiglitazone, reduced the expression of DNA-damage inducible transcript 3 (Ddit3), growth arrest and DNA-damage inducible protein 34 (Ppp1r15a) and cyclin-dependent kinase inhibitor 1a (Cdkn1a). We also monitored the levels of Pdx1 mRNA, which, however, were not different between the groups. Finally, we analyzed mRNA levels of islet hormones insulin, glucagon and somatostatin. Rosiglitazone up-regulated the levels of glucagon. All hormone mRNA levels were lowered in islets derived from *db/db *mice fed an EGCG-supplemented diet.

### Western blot analysis of pancreatic islets exposed to palmitate and EGCG *ex vivo*

**Figure 6:**
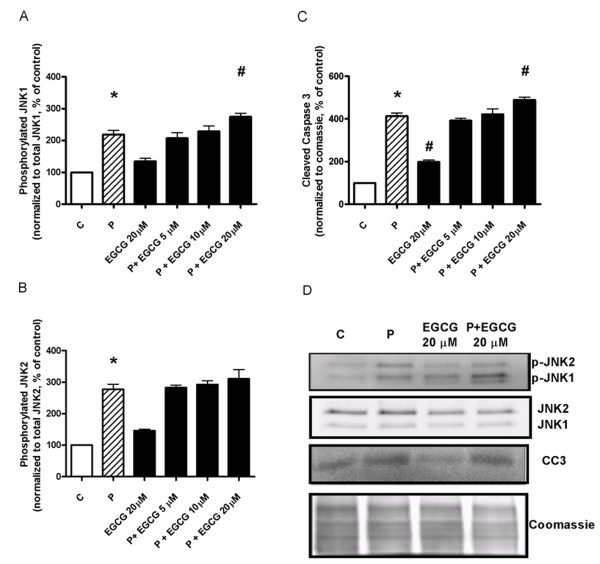
**EGCG treatment of isolated islets of Langerhans *ex vivo *fails to prevent palmitate-induced phosphorylation of JNK and formation of cleaved caspase 3**. Pancreatic islets were isolated from lean C57Bl/6J mice and exposed *ex vivo *for 24 hours in the absence or presence of 0.5 mM palmitate with or without 5, 10 or 20 μM EGCG. Western blot analyses for phosphorylation of JNK1 (**A**), JNK2 (**B**) and formation of the apoptosis marker cleaved caspase 3 (**C**). Bars represent mean ± SEM for 5 different islet preparations in each group. * denotes *P *< 0.05 for a palmitate effect, and # denotes *P *< 0.05 for an EGCG effect using one-way ANOVA in conjunction with Dunnett's multiple comparison test. Representative blots for phosphorylated JNK1 (p-JNK1), phosphorylated JNK2 (p-JNK2) and cleaved caspase 3 (CC3) are shown in **D**.

The effects of EGCG alone and on palmitate-induced cytotoxicity were investigated by measuring JNK phosphorylation and cleaved caspase 3 in isolated pancreatic islets exposed to palmitate and EGCG *ex vivo*. After 24 hours, palmitate induced a 2 to 3-fold increase in JNK1 and JNK2 phosphorylation, which was accompanied with a 3-fold induction of the cleaved form of caspase 3 (Figure 6) thus reflecting apoptosis. Neither 5 nor 10 μM of EGCG had any effect on palmitate-induced cytotoxicity, while 20 μM of EGCG caused a slight increase in JNK phosphorylation and cleaved caspase 3, both in the absence or presence of palmitate (Figure 6).

### Effect of palmitate and EGCG in vitro in MIN6 cells

**Figure 7:**
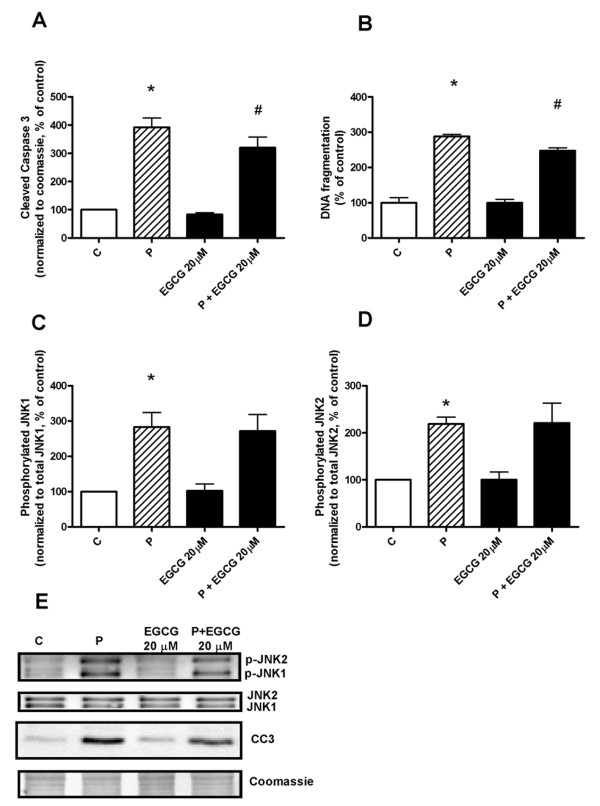
**EGCG in vitro partially protects MIN6 cells from palmitate-induced apoptosis but does not influence palmitate-induced phosphorylation of JNK**. MIN6 cells were treated with 0.5 mM palmitate in the absence or presence of 20 μM EGCG for 48 hours. Western blot analysis of formation of the apoptosis marker cleaved caspase 3 (**A**), phosphorylation of JNK1 (**C**) and JNK2 (**D**). DNA fragmentation (**B**) was measured with an ELISA against cytoplasmic oligonucleosomes. Bars represent mean ± SEM for 4 different cell preparations in each group. * denotes *P *< 0.05 for a palmitate effect, and # denotes *P *< 0.05 for an EGCG effect using one-way ANOVA in conjunction with Dunnett's multiple comparison test. Representative blots for phosphorylated JNK1 (p-JNK1), phosphorylated JNK2 (p-JNK2) and cleaved caspase 3 (CC3) are shown in **E**.

The effect of EGCG alone or in combination with palmitate was also investigated in vitro in clonal pancreatic MIN6 cells. Analysis of mRNA expression in MIN6 cells after exposure to EGCG, in the absence or presence of 0.5 mM of palmitate, for 48 hours confirmed most of the data seen in islets derived from *db/db *mice fed a diet supplemented with EGCG (Table 1). While EGCG per se was neutral, the compound reduced palmitate-induced expression of ER stress-associated genes Ddit3 and Ppp1r15a, as well as palmitate-induced expression of CPT-1. EGCG did not affect expression of Cdkn1a, Glut-2 or glucokinase but exacerbated the reduction of insulin-1 mRNA seen after palmitate treatment. EGCG alone increased cell viability in MIN6 cells (data not shown) and reduced palmitate-induced toxicity as evidenced by reduction in formation of cleaved caspase 3 and DNA fragmentation (Figure 7A and 7B), suggesting EGCG protection against palmitate-induced apoptosis. However, as with primary islets, EGCG was unable to influence palmitate-induced activation of JNK signaling (Figure 7C and 7D).

## Discussion

In this study pre-diabetic *db/db *mice were given dietary supplementation of EGCG or rosiglitazone. After 10 weeks of diet treatment mice treated with EGCG or rosiglitazone had improved glucose tolerance in an OGTT. Islet architecture was also preserved in mice receiving any of the two compounds. Our findings suggest that EGCG and rosiglitazone treatment may have attenuated death of β cells in the *db/db *mouse. In this strain of diabetic mice, EGCG and rosiglitazone treatment was associated with increases in insulin content and with preservation of islet and β cell architecture and granulation. Similar results in morphology and granulation have been observed in other animal models of diabetes when treated with rosiglitazone [16]. The results of our study are also in line with published data, which suggest that EGCG preserves and protects the islets [7,8]. However, our data also show that EGCG has a limited effect *ex vivo *with regards to its ability to protect from palmitate-induced cytotoxicity. At a high concentration in vitro it can even display cytotoxic effects to isolated islets of Langerhans, which are in concordance with at least one previous study [10]. Therefore, it is likely that dietary EGCG exerts its antidiabetic activity both through a reduction of insulin resistance and an increase in glucose-induced insulin secretion via protection of functional β cell mass. Euglycemic-hyperinsulinemic clamp studies for the determination of endogenous glucose production, gluconeogenesis and glycogenolysis will provide further understanding on the relative importance of improved insulin sensitivity and β cell function in the EGCG action.

We investigated mRNA levels in islet isolated from control mice and mice treated with either EGCG or rosiglitazone diet supplementation. Data shows that EGCG, like rosiglitazone, lowers islet expression levels of the mitochondrial fatty acid transporter L-Cpt-1. It is possible that reduced L-Cpt-1 expression is a consequence of lowered circulation fatty acid levels [17], as has been observed after EGCG and rosiglitazone feeding of *db/db *mice [18]. Reduced L-Cpt-1 expression would positively affect glucose-induced insulin secretion [19]. Our data do not provide support for enhanced glucose sensing after EGCG or rosiglitazone treatment, since neither the expression of the glucose transporter Glut-2, nor the glycolysis rate limiting enzyme glucokinase, was affected. ER stress is associated with both insulin resistance and pancreatic β cell dysfunction in several obese animal models of type 2 diabetes [14,15,20], of which the *db/db *mouse is one. Furthermore, increased expression of ER stress markers has been reported from some [14], but not all [21], *post mortem *examinations of human islets obtained from type 2 diabetic patients. Among the various ER stress markers, Ddit3 (also known as CHOP or GADD153) links ER stress to cellular apoptosis and, conversely, Ddit3 deletion reduces oxidative stress, improves β cell function and ameliorates β cell apoptosis in *db/db *mice [15]. Thus, our observation that dietary supplementation with EGCG reduces islet Ddit3 expression and its downstream target Ppp1r15a, as well as Cdkn1a, provides a plausible mechanistic explanation for the preserved islet morphology seen in the EGCG group. These findings were also true in MIN6 cells in vitro and can account for the cytoprotective effect of EGCG against palmitate-induced apoptosis seen in this cell line. From our data, it is also evident that the antidiabetic effect of rosiglitazone could not be attributed to reduced expression of Ddit3, Ppp1r15a or Cdkn1a. The mRNA levels of islet hormones were unexpectedly lowered in the EGCG group compared to the control group. Given the clear antidiabetic phenotype incurred by EGCG treatment, these results are surprising and we have not performed experiments to address the reason behind.

Antidiabetic properties of green tea have been known for some time. In the 1980s, it was reported that the green tea constituent EGCG protects pancreatic islets from alloxan by inducing restoration of blood glucose concentrations and by promoting β cell regeneration in the islets of alloxan-treated rats [22-24]. Furthermore, EGCG was reported to stimulate insulin secretion and to have insulin-like activity [25-27]. In previous work evaluating the effect of EGCG against diabetes induced in rats by the β cell toxin STZ, EGCG restored the diabetic state to normal as in other reports [23,24,28]. More recently, green tea and green tea extracts were demonstrated to beneficially modify glucose metabolism in experimental models of type 2 diabetes [29,30]. Two previous *in vivo *studies have suggested a glucose-lowering effect of EGCG [18,31]. In one of these [31], EGCG was injected into lean and obese Zucker rats ensuring supra pharmacologic plasma concentrations of EGCG and resulted in markedly decreased blood glucose and insulin levels. However, it is unclear whether these observations were due to a direct glucose lowering effect of EGCG or an anorectic effect caused by elevated plasma concentrations of EGCG. Our study extends these findings to also demonstrate a protection and preservation of pancreatic β cell function and islet morphology. Similar to previous findings in diet-induced obesity models [32], EGCG supplementation in *db/db *mice did not influence food intake. Thus, we can exclude that EGCG enhanced glucose tolerance simply by reducing food intake.

## Conclusions

In conclusion, this study demonstrates that a pharmacological dose of the green tea catechin, EGCG, possesses pronounced antidiabetic efficacy *in vivo *- comparable to the effect seen with rosiglitazone-in a mouse model of type 2 diabetes. The effects of EGCG are at least partially mediated via reduced insulin resistance and enhanced pancreatic islet function, the latter involving reduction in ER stress markers. The results also indicate that total plasma EGCG levels shown to be efficacious in mice and rats can be reached by dietary supplementation of EGCG. Therefore, our results imply that treatment of humans with type 2 diabetes with purified EGCG could be a way to confer β cell protection. This hypothesis should be investigated in randomized placebo-controlled trials.

## Competing interests

Duality of interest: Dr. Swen Wolfram is employed by DSM Nutritional Products Ltd. DSM Nutritional Products is a supplier of vitamins, carotenoids and other chemicals to the feed, food, pharmaceutical and personal care industries. All other authors have no competing interest to disclose.

## Authors' contributions

HO planned the study, conducted *in vivo *experiments, isolated islets and made all analyses with isolated islets, performed data analysis, interpreted data and drafted the manuscript. NG assisted in the experiments, made all the experiments and data analysis with MIN6 cells and drafted the manuscript. SW planned the study, provided diets and interpreted data. NK performed histological analysis. ÅS conceived the hypothesis, planned the study, interpreted data and drafted the manuscript. All authors read and approved the final version of the manuscript.
